# Clinically Meaningful Fatigue and Depression Are Associated with Sarcopenia in Patients with Non-Alcoholic Fatty Liver Disease

**DOI:** 10.3390/jpm13060932

**Published:** 2023-05-31

**Authors:** Anna F. Sheptulina, Adel A. Yafarova, Julia A. Golubeva, Elvira M. Mamutova, Anton R. Kiselev, Oxana M. Drapkina

**Affiliations:** 1 National Medical Research Center for Therapy and Preventive Medicine, Moscow 101990, Russia; sheptulina.anna@gmail.com (A.F.S.);; 2 Department of Therapy and Preventive Medicine, A.I. Evdokimov Moscow State University of Medicine and Dentistry, Moscow 127473, Russia

**Keywords:** non-alcoholic fatty liver disease, sarcopenia, fatigue, depression

## Abstract

Background: Sarcopenia is thought to be related to an increased risk of non-alcoholic steatohepatitis and advanced liver fibrosis. Our cross-sectional single-center study was designed to analyze the prevalence of sarcopenia in patients with NAFLD and possible influencing factors. Methods: A survey on the presence of sarcopenia, fatigue, anxiety, and depression, along with a quality-of-life (QoL) assessment, was forwarded by email to 189 outpatients. Demographics, anthropometric and clinical data (laboratory test results and abdomen complete ultrasound protocol), performed within 2–4 weeks prior to the enrollment, were obtained. Results: Sarcopenia (defined as SARC-F score ≥ 4) was identified in 17 (15.7%) patients, all of them (100%) females, with median age (interquartile range) 56 (51–64) years. These patients had a poorer metabolic state (greater values of waist and hip circumferences, body mass index, and HOMA-IR) and significantly poorer QoL, specifically, regarding the physical component of health, compared with NAFLD patients without sarcopenia. Multivariate analysis showed that depression (OR = 1.25, 95% CI: 1.02–1.53, *p* = 0.035) and clinically meaningful fatigue (OR = 1.14, 95% CI: 1.04–1.26, *p* = 0.008) were the factors independently associated with sarcopenia in patients with NAFLD. Conclusion: Sarcopenia is associated with depression and fatigue rather than with the severity of liver disease alone and may negatively affect QoL in patients with NAFLD.

## 1. Introduction

Nowadays, non-alcoholic fatty liver disease (NAFLD) is considered to be the most prevalent chronic liver disease worldwide [1]. Accumulated data indicate that NAFLD represents a complex multifactorial entirety associated with metabolic disorders and dysfunction of various organs [2,3], in particular, obesity, cardiovascular diseases, type-2 diabetes mellitus [4], and central-nervous-system disorders [5,6]. These conditions may affect a patient’s quality of life (QoL), social adaptiveness, and ability to work [7].

The other noteworthy issue in NAFLD is fatigue, which is believed to be just as frustrating as in patients with primary biliary cholangitis [8]. Although the pathogenesis of fatigue in NAFLD is considered to be complex and remains poorly understood, potential mechanisms may include extrahepatic factors, such as adipose tissue inflammation. Moreover, given the accumulated evidence on the possible role of skeletal muscles in the development and progression of NAFLD, as well as their involvement in energy homeostasis, it can be assumed that skeletal-muscle disorders (myosteatosis and sarcopenia) could also be important contributing factors.

Myosteatosis, defined as skeletal-muscle fat infiltration, is common in NAFLD patients [9]. It is suggested that myosteatosis may result in sarcopenia, a syndrome characterized by progressive and generalized loss of muscle mass, strength and function. The latter was formerly considered as a feature of aging, but actually may occur at any age due to multiple causes, such as insufficient physical activity, unhealthy diet, and chronic noncommunicable diseases. Sarcopenia is known to be related to a risk of adverse outcomes, namely, poor QoL, physical disability, and death [10,11]. Furthermore, quality and quantity of muscles are recognized as important prognostic factors determining the probability of non-alcoholic steatohepatitis (NASH) and advanced liver fibrosis [10].

Currently, the most effective and recognized treatment for NAFLD is lifestyle modification through physical activity and diet. However, due to the established relationship between NAFLD, depression, low assiduousness and high neuroticism, as well as the low willingness of patients with NAFLD to follow physicians’ recommendations on the lifestyle modification strategies, achieving the goals of NAFLD therapy can be quite difficult [12]. Sarcopenia may be an additional factor preventing increased physical activity and lifestyle changes. The adverse effect of this condition could be associated with its direct consequences (a decrease in physical performance; an inability to exercise) as well as with its indirect effects—possibly, through participation in the development of fatigue and a contribution to the deterioration of mental health. In fact, K.V. Chang et al. (2017) demonstrated that sarcopenia positively correlated with depression, and this relationship remained significant after adjusting for potential confounding factors, namely, gender, age, physical activity, and cognitive ability [13]. As far as we know, there are currently no published studies evaluating the association between sarcopenia, mood disorders, and fatigue in patients with NAFLD.

The aim of this study was to examine the prevalence of sarcopenia in NAFLD patients, as well as to assess its relationship with clinically significant fatigue, mood disorders, and other relevant factors due to their adverse effect on QoL, long-term prognosis, and effectiveness of therapy in this category of patients.

## 2. Materials and Methods

### 2.1. Study Participants

The cross-sectional single-center study was conducted at the Clinical and Diagnostic Division of the National Medical Research Center for Therapy and Preventive Medicine. Based on the known probability of depression and fatigue in patients with NAFLD [14,15,16], we calculated that the total sample size in this study should be not less than 101 at an alpha of 0.05 and a power of 0.80 [17]. During the sample-size calculation, we took into consideration unequal sample sizes (with approximate allocation ratio 4.5:1) based on the available data on the prevalence of sarcopenia in patients with NAFLD [18,19]. Consistently and prospectively, all adult patients with NAFLD who sought medical care at the Clinical and Diagnostic Division from February to July 2020 and were eligible for present study were included. The study was carried out in accordance with the Helsinki-Declaration guidelines. Study protocol was approved by the local Research Ethics Committee of the Institution (protocol No. 01-03/20 of 23 January 2020). All included patients signed informed-consent form.

Overall, 189 patients with NAFLD participated in an e-mail survey. Inclusion criteria were as follows: (1) age 18 to 75 years; (2) Fatty Liver Index (FLI) values ≥ 60; (3) ultrasonographic signs characteristic of fatty liver disease (specified further); (4) absence of concomitant liver disease (based on serologic testing for hepatitis B and hepatitis C virus infections, as well as serum γ-globulin and immunoglobulin G levels); and (5) a recent outpatient visit requiring laboratory tests and ultrasound assessment (2–4 weeks before enrollment).

Exclusion criteria were set as follows: concomitant liver disease (based on serologic testing for hepatitis B and hepatitis C virus infections, as well as serum γ-globulin and immunoglobulin G levels), significant consumption of the alcohol (>20 g/day), morbid obesity, acute infectious diseases or exacerbation of chronic noncommunicable diseases (within four weeks prior to inclusion), advanced chronic kidney disease (GFR < 30 mL/min/1.73 m^2^), mental disorders, limiting cooperation with study personnel, a history of malignant neoplasms of any localization, lower extremity fractures within 6 months before the study with persistent negative impact on the functional state, any clinically significant disorders or diseases that prevent movement and self-care, pregnancy and breastfeeding, as well as the lack of consent.

It is worth noting that the presence of type-2 diabetes mellites (T2DM) was also set as exclusion criteria in our study, in order to eliminate the possible influence of T2DM on the prevalence of sarcopenia in patients with NAFLD due to the increasing evidence of bidirectional relationship between sarcopenia and T2DM [20,21].

After signing the informed-consent form, patients were asked to fill in the questionnaires for assessment of fatigue, quality of life (QoL), depression, anxiety, or sarcopenia. When completing the questionnaires, patients were asked to describe their well-being over the past month. The time required to complete the questionnaires was approximately 45 min. Demographic, anthropometric, and clinical data were obtained from patient medical records.

### 2.2. Assessments

#### 2.2.1. Assessment of Fatty Liver Disease

Non-alcoholic fatty liver disease was diagnosed on the basis of abdominal ultrasonography signs, including brightness of liver parenchyma, liver-to-kidney contrast, impaired visualization of deeper liver parenchyma and the diaphragm, as well as decreased conspicuity of hepatic vessels.

In addition, Fatty Liver Index (FLI) was calculated using the following expression [22]:

FLI = (e^0.953 × ln (triglycerides) + 0.139 × BMI + 0.718 × ln (GGT) + 0.053 × waist circumference − 15.745^)/(1 + e^0.953 × ln (triglycerides) + 0.139 × BMI + 0.718 × ln (GGT) + 0.053 × waist circumference − 15.745^) × 100,

where BMI is body mass index and GGT is gamma-glutamyl transferase.

Cutoff value of FLI ≥ 60 was used to rule in fatty liver disease (positive likelihood ratio = 4.3) [22].

We used fibrosis-4 (FIB-4) score to exclude advanced liver fibrosis. It was calculated using the following expression [23]:

FIB-4 = age (years) × AST [U/L]/(platelets [10^9^/L] × (ALT [U/L])^1/2^),

where AST is aspartate aminotransferase and ALT is alanine aminotransferase.

The negative predictive value of FIB-4 scores < 1.3 for the exclusion of advanced liver fibrosis was >90% [23].

#### 2.2.2. Assessment of Anxiety and Depression

We used the Hospital Anxiety and Depression Scale (HADS) to assess the presence of anxiety and depression. It was developed specifically for evaluating anxiety and depression in patients without psychiatric disorders, as well as in the general population [24]. This scale proved to be effective and reliable diagnostic tool in patients with chronic liver diseases of different etiology [14]. We calculated composite scores separately for depression (HADS-D) and anxiety (HADS-A). Individuals were assumed to have depression/anxiety if they had a HADS-D/HADS-A score of 8 or greater [25].

#### 2.2.3. Assessment of Fatigue

In order to assess fatigue, we used the Fatigue Assessment Scale (FAS). This scale measures mental and physical components of fatigue, irrespective of the presence of neuroticism or depression. The Fatigue Assessment Scale demonstrated good performance in clinical trials [26]. Recent study showed that FAS is a reliable tool to measure fatigue in patients with chronic hepatitis C [27]. According to Bhandari K et al. (2022), FAS may be recommended for the evaluation of this symptom in patients with chronic liver diseases [28]. Fatigue Assessment Scale includes 10 multiple-choice statements. The overall score ranges from 10 to 50 points. Cutoff values of ≥22 points suggest the presence of clinically significant fatigue [29].

#### 2.2.4. Assessment of Sarcopenia

In order to identify sarcopenia, we used the SARC-F questionnaire. This questionnaire is recommended by the European Working Group on Sarcopenia in Older People (EWGSOP) for the selection of patients with signs and symptoms specific for sarcopenia [30]. It allows to evaluate five components: strength, walking ability, limitations in rising from a chair and climbing the stairs, along with a history of falls. The overall score ranges from 0 to 10. Cutoff values of ≥4 suggest the presence of sarcopenia and indicate the need for further evaluation.

#### 2.2.5. Quality-of-Life Assessment

We used the 36-Item Short Form Health Survey (SF-36) to assess the QoL. This questionnaire represents a reliable tool for the evaluation of QoL both in the general population and in patients with various chronic diseases. In addition, it has already shown good performance in patients with NAFLD [31]. The 36-Item Short Form Health Survey is comprised of eight subscales grouped into two summary scores. They are physical functioning (PF), general health (GH), role limitations due to physical health problems (RP), and bodily pain (BP) together constituting physical component summary (PCS), and vitality (VT), role limitations due to personal or emotional problems (RE), social functioning (SF), and mental health (MH) together comprising mental component summary (MCS). The scores may range from 0 to 100, with higher scores being characteristic of better health [32].

### 2.3. Statistical Analysis

Based on the known probability of depression and fatigue in patients with NAFLD [14,15,16], we calculated that the total sample size in this study should be not less than 101 at an alpha of 0.05 and a power of 0.80 [17]. During the sample-size calculation, we took into consideration unequal sample sizes (with approximate allocation ratio 4.5:1) based on the available data on the prevalence of sarcopenia in patients with NAFLD [18,19]. Continuous variables are presented as medians and interquartile ranges (IQR), whereas categorial variables are reported as frequencies and percentages. Mann–Whitney U test and Pearson’s chi-squared test (χ2) were used for comparisons between two groups. In order to identify independent risk factors for the presence of sarcopenia, we employed the logistic regression analysis. A *p*-value of <0.05 was considered statistically significant. Data were analyzed using IBM SPSS statistics software version 28.0 (IBM Corp., Armonk, NY, USA).

## 3. Results

Overall, 108 out of 189 (57.1%) eligible NAFLD patients with complete data comprised the study population. Table 1 summarizes the main characteristics of the study subjects. The median age was 49.5 years (IQR, 41.3–58.8). Seventy-one patients (65.7%) were obese, and only four patients (3.7%) had normal BMI values. Overall, the blood biochemistry profile of included patients was consistent with the metabolic disturbances common to metabolic syndrome and specifically to NAFLD.

Clinically significant fatigue (defined as FAS score ≥ 22) was present in 49.1% of patients. Twenty-two patients (20.4%) had anxiety scores of ≥8 (median score (IQR): 9 (8–10.8)), and 21 patients (19.4%) had abnormal depression scores (median score (IQR): 10 (8–11.5)). Sarcopenia (SARC-F score ≥ 4) was identified in 17 (15.7%) patients with NAFLD.

Table 2 presents parameters significantly different between the subgroups of NAFLD patients depending on the presence of sarcopenia. There were no significant differences in blood biochemical parameters (AST, ALT, total bilirubin, total cholesterol, uric acid, C-reactive protein, etc.) between the subgroups, with the exclusion of serum creatinine. This parameter was significantly higher in NAFLD patients without sarcopenia (*p* = 0.032). At the same time, GGT, AST activity, and concentration of triglycerides tended to be lower in patients with NAFLD without sarcopenia (*p* = 0.058, *p* = 0.096, and *p* = 0.054, respectively). In addition, patients with NAFLD without sarcopenia had lower HOMA-IR values compared to patients with NAFLD and sarcopenia (*p* = 0.008), though the proportion of subjects with HOMA-IR > 2.7 was similar in both groups. Lastly, there were no statistically significant differences between the subgroups concerning the share of patients with FIB-4 < 1.3 (14 (82.4%) patients in NAFLD with sarcopenia subgroup vs. 74 (81.3%) patients in NAFLD without sarcopenia subgroup, *p* = 0.839), and the share of patients with anxiety (3 (17.6%) patients in the NAFLD with sarcopenia subgroup vs. 19 (20.9%) patients in the NAFLD without sarcopenia subgroup, *p* = 0.761).

**Figure 1:**
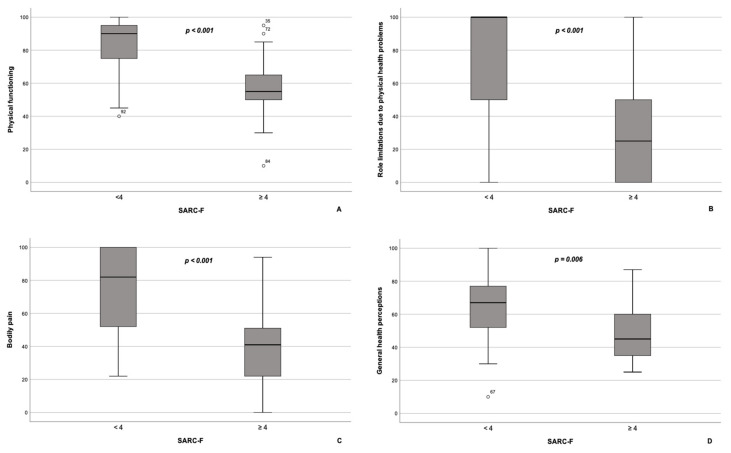
Box plot of the subscales comprising the physical component summary of the SF-36 questionnaire for patients with NAFLD with (*n* = 17) and without sarcopenia (*n* = 91). Patients with NAFLD and sarcopenia had significantly lower scores on physical functioning (**A**), role limitations due to physical health problems (**B**), bodily pain (**C**), and general health perceptions (**D**) subscales compared to patients with NAFLD without sarcopenia. The line through the middle of each box represents the median. The length of the box, thus, represents the interquartile range. The error bars show the minimum and maximum values of each subscale. Outliers are depicted as circles. All comparisons are performed using Mann–Whitney U test.

**Figure 2:**
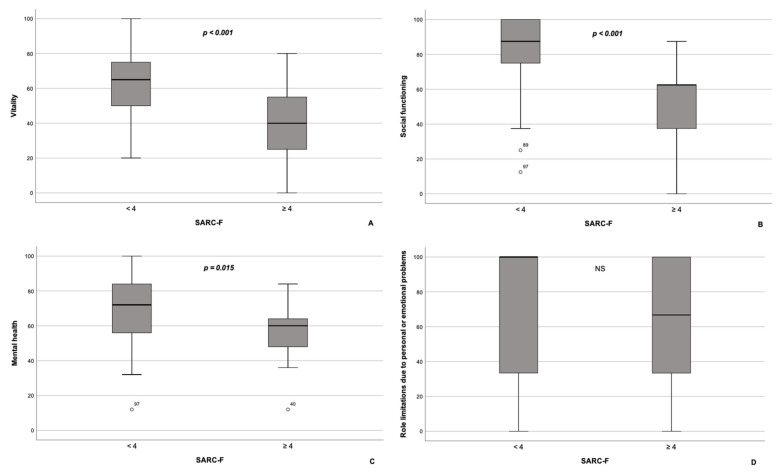
Box plot of the subscales comprising the mental component summary of the SF-36 questionnaire for patients with NAFLD with (*n* = 17) and without sarcopenia (*n* = 91). Patients with NAFLD and sarcopenia had significantly lower scores on vitality (**A**), social functioning (**B**), and mental health (**C**) subscales compared to patients with NAFLD without sarcopenia. On the contrary, scores on the role limitations due to personal or emotional problems subscale (**D**) did not differ significantly between the groups. The line through the middle of each box represents the median. The length of the box, thus, represents the interquartile range. The error bars show the minimum and maximum values of each subscale. Outliers are depicted as circles. All comparisons are performed using Mann–Whitney U test. NS—non-significant.

According to our results, PCS scores (27.6 (26.3–39.0) vs. 49.5 (44.1–56.6), *p* < 0.001), as well as the scores on all subscales comprising the PCS, namely, PF, RP, BP, and GH, were significantly lower in NAFLD patients with sarcopenia compared with NAFLD patients without sarcopenia (Figure 1A–D). Contrarywise, scores on MCS were similar between the subgroups (45.2 (37.2–51.2) in NAFLD patients with sarcopenia vs. 48.8 (41.9–55.5) in NAFLD patients without sarcopenia, *p* = 0.092). As for subscales, constituents of MCS, scores on VT, SF, and MH subscales, were significantly lower in patients with NAFLD and sarcopenia compared with the subgroup of NAFLD patients without sarcopenia (Figure 2A–C), whereas RE scores did not differ significantly between the groups (*p* = 0.370; Figure 2D). 

Based on stepwise regression analysis, the only factors independently associated with the presence of sarcopenia (defined as SARC-F score ≥ 4) in NAFLD patients were clinically meaningful fatigue (odds ratio (OR), 95% confidence interval (95 % CI): 1.14, 1.035–1.259; *p* = 0.008) and depression (OR, 95% CI: = 1.248, 1.016–1.534; *p* = 0.035).

## 4. Discussion

Currently, NAFLD has reached epidemic proportions. The pathophysiology of NAFLD seems to be complex and multifactorial, involving many organ systems, although the role of skeletal muscles in this process was largely ignored for a long period of time. Increasing evidence suggests that skeletal muscle and the muscle–liver axis are central to the pathogenic NAFLD cascade.

In our cross-sectional single-center study, the prevalence of sarcopenia, defined as SARC-F score ≥ 4, in NAFLD patients was 15.7%. As the study population comprised all consecutive patients who visited the Clinical and Diagnostic Division from February to July 2020 and met the inclusion criteria, the prevalence of sarcopenia in our study was consistent with that of the NAFLD patient population. In particular, our data are similar to those presented in a Chinese cross-sectional observational study including 578 participants, 154 of whom were diagnosed with NAFLD. In accordance with the results of a dual-energy X-ray absorptiometry (DXA), the prevalence of sarcopenia in NAFLD patients was 16.2%, and it appeared to be higher in men than in women (20.0% vs. 15.3%, respectively) [18]. In contrast, all patients diagnosed with sarcopenia in our study were women, and this discrepancy in data may be due to the method used to detect sarcopenia, as well as racial differences between the studied populations. In addition, our data are consistent with those of B.K. Koo et al. (2017), which indicate that the prevalence of sarcopenia was 17.9% among patients with hepatic steatosis (*n* = 117) and 35% among those with NASH (*n* = 123) [19]. Based on the results of a retrospective analysis of computed tomography (CT) data, sarcopenia was more common in patients with biopsy-proven NAFLD (*n* = 83) compared to controls (patients who underwent a CT of the abdomen for the detection of renal stones, *n* = 83). In particular, patients with NAFLD had decreased paravertebral muscles’ mass (psoas and lumbar paraspinal muscles) compared to controls (*p* < 0.001) [33].

Overall, the prevalence of sarcopenia among patients with NAFLD remains largely unknown. This may be due to the fact that clear diagnostic criteria for sarcopenia, especially in obese patients, are lacking [11]. In addition, there is no consensus on threshold values for various diagnostic tools for measuring muscle function and quantity, such as grip strength, skeletal-muscle mass index (SMI), or appendicular skeletal-muscle mass index (ASMI), etc. Finally, the application of different methods to assess muscle quality and quantity can complicate the comparison of the results from different studies.

In our study, the prevalence of sarcopenia in NAFLD patients increased with age and BMI values. These findings comply with published data on the higher prevalence of sarcopenia in older patients with NAFLD [34], and with data by Donini et al. (2022) on the higher likelihood of obese patients to develop sarcopenia at any age [11]. Development of sarcopenia in overweight/obese subjects could be explained by the following mechanisms. First, obesity can serve as independent factor resulting in the muscle-mass loss and deterioration of muscle function. This may be explained by the adverse effect of metabolic disorders associated with the excess accumulation of adipose tissue in the body [11]. This hypothesis is also supported by our results, indicating that patients with NAFLD and sarcopenia experience a worse metabolic condition, implied by the higher values of waist and hip circumferences, insulin levels, as well as HOMA-IR scores. Overall, blood biochemical parameters were similar between the subgroups of NAFLD patients with and without sarcopenia, albeit GGT, AST, and triglyceride levels were somewhat higher in patients with sarcopenia. These data are in accordance with results by B.K. Koo et al. (2017), who demonstrated that NAFLD subjects with sarcopenia were in a poorer metabolic condition than those without it: they had higher values of HOMA-IR, BMI, waist circumference, and serum concentrations of AST, GGT and high-sensitivity C-reactive protein [19].

Higher prevalence of chronic noncommunicable diseases known to have a negative impact on the metabolism of muscle tissue could constitute another mechanism explaining the development of sarcopenia in overweight/obese patients. Finally, a sedentary lifestyle, being a primary cause or a consequence of sarcopenia and/or obesity, may be of major relevance [11]. In this regard, it is important to mention the finding on the differences in serum creatinine between the subgroups of NAFLD patients with and without sarcopenia. It is well known that serum creatinine is a reliable marker of muscle mass and kidney function. In our study, serum creatinine levels were lower in patients with NAFLD and sarcopenia. Although we could not exclude the well-known sex-specific difference in serum creatinine levels (women constituting the NAFLD group with sarcopenia in this study usually have lower serum creatinine levels than men) [35], this difference could be due to presence of sarcopenia, per se. This finding is consistent with the hypothesis that serum creatinine in combination with cystatin C can serve as a surrogate marker of sarcopenia [36,37]. It may also be an important prognostic marker in this category of patients. In particular, C. Thongprayoon et al. (2017) established that a decrease in the serum concentration of creatinine, probably due to the presence of sarcopenia, was accompanied by an increase in mortality rate [38]. 

According to HADS and FAS scores, depression and fatigue were more common in patients with NAFLD and sarcopenia. On the one hand, these findings are consistent with the data published by Gu Y. et al. (2022), indicating an increased prevalence of depression in patients with NAFLD (pooled OR, 95% CI: 1.13, 1.03–1.24, *p* = 0.007) [15], as well as with the data obtained by Chang KV. et al. (2017), which describe a significant direct relationship between depression and sarcopenia (adjusted OR, 95% CI: 1.821, 1.160–2.859) [13]. On the other hand, they demonstrate the association between two pathological conditions, fatigue and sarcopenia, implying an important role of skeletal muscles (as organs involved in energy homeostasis) in the pathogenesis of fatigue in NAFLD. This may be of particular importance when considering the findings published by J. Newton et al. (2008) [8]. The authors concluded that neither insulin resistance nor severity of the liver disease were associated with the fatigue in NAFLD, thereby questioning the critical role of liver disease per se in the pathophysiology of this troubling symptom. In turn, J. Newton et al. (2008) suggested that impaired physical activity may be the key to understanding the origin of fatigue in NAFLD [8]. However, further prospective longitudinal studies are needed to confirm the association between fatigue and sarcopenia in NAFLD.

Finally, according to the results of our study, the subgroup of NAFLD patients with sarcopenia reported substantially poorer QoL compared to the subgroup of NAFLD patients without sarcopenia, specifically, regarding the physical component of health, which complies with previously published data [39]. However, as far as we know the association of sarcopenia with reduced QoL among patients with NAFLD has not been previously reported. 

## 5. Limitations

Our study has several limitations. First, in our study, we diagnosed sarcopenia based exclusively on the SARC-F questionnaire, which is currently recommended to be used as a screening tool for sarcopenia. According to the European Consensus, this questionnaire has a high specificity, but moderate sensitivity in defining sarcopenia, which means that it is able to detect mostly severe sarcopenia (characterized by the loss of muscle mass and strength along with the deterioration of muscle function). We did not use any objective method to reflect the presence of sarcopenia; consequently, more thorough research including tests to assess muscle mass (i.e., bioelectrical impedance analysis, dual energy X-ray absorptiometry, magnetic resonance imaging, or computed tomography), muscle strength (i.e., grip strength or chair stand test), and muscle function (i.e., short physical performance battery or gait speed) may provide more accurate data on the prevalence of sarcopenia in NAFLD. Second, the study had a cross-sectional design, making it impossible to determine whether the presence of sarcopenia caused clinically meaningful fatigue, or vice versa. Despite these limitations, our research revealed an association between sarcopenia, fatigue and depression in patients with NAFLD, and pointed out that the presence of sarcopenia could contribute to the deterioration of QoL in patients with NAFLD. Finally, the collected data provide a different perspective on the problem of fatigue in NAFLD. It may seem that insufficient physical activity may be the cause, rather than a consequence, of the fatigue.

## 6. Conclusions

In conclusion, we suggest that sarcopenia is a widespread condition in patients with NAFLD. It proved to be associated with clinically meaningful fatigue and depression and has an adverse impact on the QoL of NAFLD patients. Due to both direct and indirect consequences, sarcopenia may be considered a substantial factor for the development and progression of NAFLD, further hindering the achievement of therapy goals.

## Figures and Tables

**Table 1:** Demographic, anthropometric, and biochemical parameters of 108 NAFLD patients who completed the survey.

Parameter	NAFLD Patients (*n* = 108)
Gender: female, *n* (%)	68 (63)
Age, years	49.5 (41.3–58.8)
BMI, kg/m^2^	31 (28.7–34.5)
Normal weight (BMI < 25 kg/m^2^), *n* (%)	4 (3.7)
Overweight (25 ≤ BMI < 30 kg/m^2^), *n* (%)	33 (30.6)
Obese (BMI ≥ 30 kg/m^2^), *n* (%)	71 (65.7)
Waist circumference, cm	101.7 (95–109)
Hip circumference, cm	112 (106–120)
Platelet count, 10^9^/L	254 (207–289)
WBC, 10^9^/L	5.8 (4.6–6.9)
ALT, IU/L	23 (17–33)
AST, IU/L	21 (17–26)
GGT, IU/L	30 (21.8–49)
Total bilirubin, mg/dL	0.64 (0.47–0.88)
Glucose, mmol/L	5.7 (5.2–6.2)
Total cholesterol, mg/dL	201.1 (173.9–231.9)
Triglycerides, mg/dL	132.8 (98.5–194.5)
Fibrinogen, g/L	3.9 (3.6–4.3)
CRP, mg/L	1.9 (1–3.7)
Creatinine, μmol/L	75 (69–87)
Uric acid, μmol/L	362.9 (291.6–416.5)
Insulin, μIU/mL	11.1 (8.1–14.4)
HOMA-IR > 2.7, *n* (%)	47 (43.5)
TyG ≥ 8.5, *n* (%)	64 (59.3)
FIB-4 < 1.3, *n* (%)	84 (77.8)

The presented values denote frequency (%) or median (interquartile range). BMI: body mass index; ALT: alanine aminotransferase; AST: aspartate aminotransferase; CRP: C-reactive protein; FIB-4: fibrosis-4 index; GGT: gamma-glutamyl transferase; HOMA-IR: homeostasis model assessment of insulin resistance; TyG: triglyceride glucose index; WBC: white blood cell count.

**Table 2:** Parameters significantly differing between the groups of NAFLD patients with (*n* = 17) and without sarcopenia (*n* = 91).

Parameter	NAFLD Patients with SARC-F Score ≥ 4 (NAFLD with Sarcopenia, *n* = 17)	NAFLD Patients with SARC-F Score < 4 (NAFLD without Sarcopenia, *n* = 91)	*p*-Value
Gender: female, *n* (%)	17 (100)	51 (56)	0.001 ^a^
Age, years	56 (51–64)	48 (40–58)	0.012 ^b^
BMI, kg/m^2^	32.9 (30.6–36.6)	30.5 (28.5–34.2)	0.045 ^b^
Waist circumference, cm	102 (100.5–112.3)	100.5 (94–108.3)	0.043 ^b^
Hip circumference, cm	116 (113–123)	110 (105–117.4)	0.015 ^b^
Insulin, μIU/mL	13.7 (10–20.7)	10.4 (7.9–14.2)	0.032 ^b^
HOMA-IR	4.2 (2.9–5.6)	2.7 (1.9–3.5)	0.008 ^b^
Creatinine, μmol/L	72 (66.5–77)	76 (70–87)	0.032 ^b^
FAS ≥ 22, *n* (%)	13 (76.5)	40 (44)	0.014 ^a^
HADS-D ≥ 8, *n* (%)	7 (41.2)	14 (15.4)	0.014 ^a^

The presented values denote frequency (%) or median (interquartile range). HADS-A: Hospital Anxiety and Depression Scale, subscale for anxiety; HADS-D: Hospital Anxiety and Depression Scale, subscale for depression; SARC-F: strength, assistance with walking, rise from a chair, stair climbing, and history of falls. ^a^ Pearson’s chi-squared test (c2); ^b^ Mann–Whitney U test.

## Data Availability

The datasets used and analyzed in the present study are available from the corresponding author on reasonable request.
